# *In situ* preparation of fluorescent CdTe quantum dots with small thiols and hyperbranched polymers as co-stabilizers

**DOI:** 10.1186/1556-276X-9-121

**Published:** 2014-03-17

**Authors:** Yunfeng Shi, Zhimin Ma, Ningning Cui, Yanli Liu, Xiaoyu Hou, Weimin Du, Lin Liu, Tong Gangsheng

**Affiliations:** 1 School of Chemistry and Chemical Engineering, Anyang Normal University, Anyang 455000, People's Republic of China; 2 Instrumental Analysis Center, Shanghai Jiao Tong University, 800 Dongchuan Road, Shanghai 200240, People's Republic of China

**Keywords:** Hyperbranched poly (amidoamine) s, Quantum dots, Nanocomposites, Photoluminescence

## Abstract

A new strategy for *in situ* preparation of highly fluorescent CdTe quantum dots (QDs) with 3-mercaptopropionic acid (MPA) and hyperbranched poly(amidoamine)s (HPAMAM) as co-stabilizers was proposed in this paper. MPA and HPAMAM were added in turn to coordinate Cd^2+^. After adding NaHTe and further microwave irradiation, fluorescent CdTe QDs stabilized by MPA and HPAMAM were obtained. Such a strategy avoids the aftertreatment of thiol-stabilized QDs in their bioapplication and provides an opportunity for direct biomedical use of QDs due to the existence of biocompatible HPAMAM. The resulting CdTe QDs combine the mechanical, biocompatibility properties of HPAMAM and the optical, electrical properties of CdTe QDs together.

## Background

Fluorescent quantum dots (QDs) exhibit unique size and shape-dependent optical and electronic properties [1-9]. They are of great interest to many applications such as optoelectronics, photovoltaic devices, and biological labels. Developing new method to prepare QDs with controlled size and shape is always an important research area. To be now, organometallic way [10-14], aqueous route with small thiols as stabilizers [15-19], dendritic polymers [20-22] as nanoreactors and biotemplate synthesis [23] are the common methods to prepare QDs. The QDs prepared by organometallic way or aqueous route with small thiols as stabilizers usually have high quantum yield, but they need to be modified in order to be suitable for their biological application. The QDs prepared by dendritic polymers or biotemplate always has low quantum yield and broad emission spectrum. So we would like to propose a new method by which highly fluorescent CdTe QDs which can be directly used for biomedical applications can be prepared.

In this study, we used 3-mercaptopropionic acid (MPA) and hyperbranched poly(amidoamine)s (HPAMAM) as co-stabilizers to prepare highly fluorescent CdTe QDs. MPA is always used to prepare luminescent CdTe QDs in aqueous phase. HPAMAM has low cytotoxicity and can be used to gene transfection and drug delivery [24]. Consequently, by using MPA and HPAMAM as co-stabilizers, highly luminescent and biocompatible CdTe QDs can be synthesized. The resulting CdTe QDs can be directly applied to bioimaging, gene transfection, etc.

## Methods

### Materials

Amine-terminated HPAMAM was synthesized according to our previous work [25]. After endcapping by palmityl chloride, the weight average molecular weight (Mw) of HPAMAM measured by gel permeation chromatography (GPC) was about 1.1 × 10^4^ and the molecular weight polydispersity (PDI) was 2.7. CdCl_2_ · 2.5 H_2_O (99%), NaBH_4_ (96%), tellurium powder (99.999%), and methanol were purchased from Sinopharm Chemical Reagent Co., Ltd., Shanghai, China. 3-Mercaptopropionic acid (MPA, >99%) was purchased from Fluka, St. Louis, MO, USA. The ultrapure water with 18.2 MΩ · cm was used in all experiments.

### Synthesis of CdTe QDs with MPA and HPAMAM as co-stabilizers

MPA (26 μL) was added to 100 mL CdCl_2_ (0.125 mmol) aqueous solution. After stirring for several hours, pH value of the aqueous solution was adjusted to 8.2 with 1 M NaOH. Then, 120 mg HPAMAM in 2 mL water was drop-added under N_2_ atmosphere and stirred for 24 h. After deaeration with N_2_ for 15 min, 10 mL oxygen-free NaHTe solution was injected at 5°C under vigorous stirring; thus, CdTe precursor solution stabilized by MPA and HPAMAM was obtained. Then, the mixture was irradiated at different times under microwave (PreeKem, Shanghai, China, 300 W, 100°C) to get a series of samples with various colors.

### Characterization of the as-prepared CdTe QDs

pH values were measured by a Starter 3C digital pH meter, Ohaus, USA. Transmission electron microscopy (TEM), selected area electron diffraction (SAED), and elemental characterization were done on a JEOL 2010 microscope (Akishima-shi, Japan) with energy-dispersive X-ray spectrometer (EDS) at an accelerating voltage of 200 kV. X-ray powder diffraction (XRD) spectrum was taken on Rigaku Ultima III X-ray diffractometer (Shibuya-ku, Japan) operated at 40 kV voltage and 30 mA current with Cu Ka radiation. UV-visible (vis) spectra were recorded on a Varian Cary 50 UV/Vis spectrometer, Agilent Technologies, Inc., Santa Clara, CA, USA. Emission spectra were collected using a Varian Cary spectrometer. Thermogravimetric analysis (TGA) was done under nitrogen on a STA 409 PC thermal analyzer, Netzsch, Germany. The quantum yield (QY) of CdTe QDs was measured according to the methods described in [26] using rhodamine 6G as a reference standard (QY = 95%).

## Results and discussion

**Figure 1:**
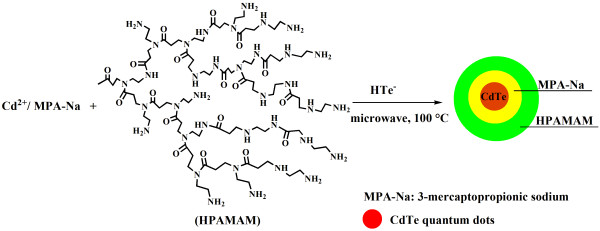
**Illustration for the facile preparation of highly luminescent CdTe QDs with MPA and HPAMAM as co**-**stabilizers.**

HPAMAM have three-dimensional topological structures, many inner cavities, and a large amount of terminal functional groups. They have low cytotoxicity and have been widely used in biomedical science, such as gene transfections and drug delivery [24]. They also can be used to prepare nanocrystals such as CdS nanocrystals, but they cannot cap the nanocrystals very compactly compared to small thiols. If nanocrystals are not capped closely, they might be unstable and tend to be oxidized. Based on this, we proposed a new strategy for preparing CdTe QDs with MPA and HPAMAM as co-stabilizers, so the resulting CdTe QDs can be coated closely and high QY can be reached. MPA and HPAMAM were added in turn to coordinate Cd^2+^. After adding NaHTe and further microwave irradiation, fluorescent CdTe QDs stabilized by MPA and HPAMAM were obtained, as illustrated in Figure 1. By preparing CdTe QDs by MPA and HPAMAM, the mechanical, biocompatibility properties of HPAMAM and the optical, electrical properties of CdTe QDs can be combined, endowing the CdTe QDs with biocompatibility.

**Figure 2:**
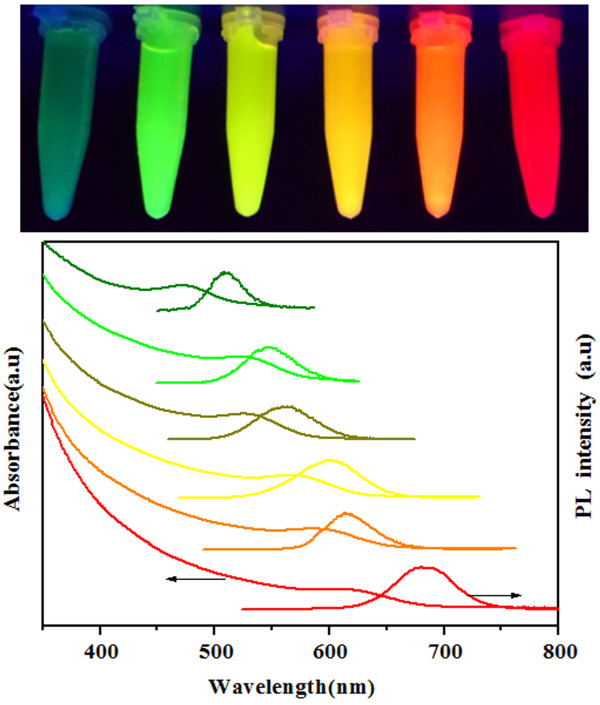
**Photograph of different**-**sized CdTe QDs and the corresponding absorption and photoluminescence spectra.** Photograph of different**-**sized CdTe QDs (stabilized by both HPAMAM and MPA) made under an UV lamp (top) and the corresponding absorption (bottom) and photoluminescence (PL) spectra (bottom). The PL emission peaks were at 509, 546, 563, 578, 605, and 629 nm, respectively.

**Figure 3:**
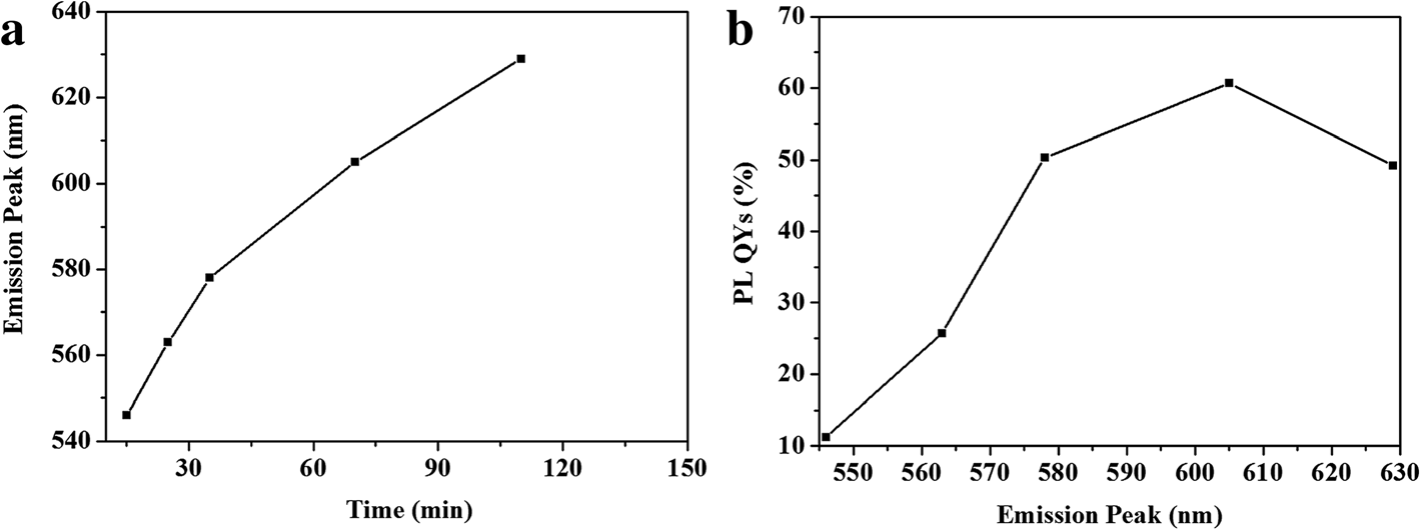
**CdTe QDs emission peak position vs. reaction time (a) and PL QYs vs. emission peak (b).** The reaction temperature was 100°C.

Figure 2 shows the photograph of different-sized CdTe QDs (stabilized by both MPA and HPAMAM) made under an UV lamp (top) and the corresponding absorption (bottom) and photoluminescence (PL) spectra (bottom). The fluorescent color of CdTe QDs under UV light changed from green to yellow orange, and red with prolonging heating time. All the absorption shoulders in the UV-vis spectra shifted to a longer wavelength during the heating treatment, indicating the growth of CdTe QDs. The maximum peak of PL emission also shows red shift, and this can also be seen in Figure 3a. While increasing the heating time, the QY of CdTe QDs increased significantly. The QY increased markedly from 11.2% at 15 min to a maximum value of 60.8% at 70 min. Further heating resulted in a slight decrease of QY, as shown in Figure 3b. The sizes of CdTe QDs can be estimated from the absorption peaks using Peng's empirical formula [27]. From the absorption peaks, the Peng's empirical formula predicts that the diameter of CdTe QDs is from 2.8 to 3.6 nm.

**Figure 4:**
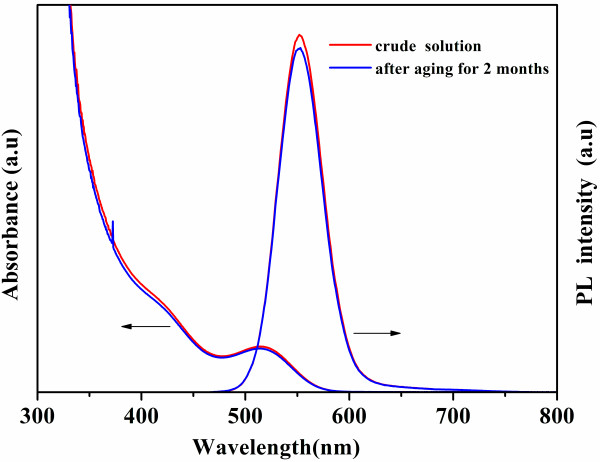
**The absorption and emission spectra of CdTe aqueous solution before and after being aged for 2 months.** The absorption peak of CdTe QDs is 515 nm.

The stability of CdTe QDs is important for their application, so we kept some samples taken at different irradiation times to investigate their stability. Figure 4 shows the absorption and emission spectra of CdTe aqueous solution before and after being aged for 2 months. After being kept for 2 months, the absorption and photoluminescence spectra of CdTe QDs (the excitonic absorption peak at 515 nm) had only slight changes, indicating the high stability of CdTe QDs.

**Figure 5:**
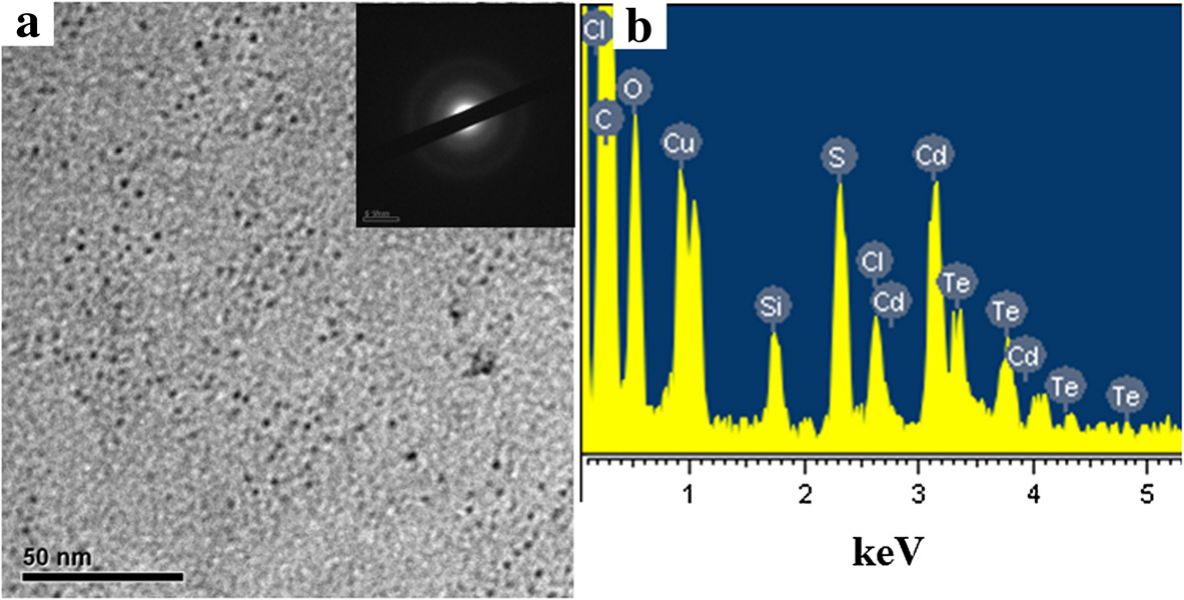
**TEM image and EDS spectrum of CdTe QDs. (a)** TEM image (inset, the corresponding SAED pattern) and **(b)** EDS spectrum of CdTe QDs stabilized both by MPA and HPAMAM (the excitonic absorption peak at 589 nm).

The morphology of CdTe QDs (the excitonic absorption peak at 589 nm) was characterized by TEM, as shown in Figure 5. From the TEM image, we can see the size of CdTe QDs is about 3.5 nm, and the size is quite uniform. The SAED pattern inside Figure 4a shows that the synthesized fluorescent nanoparticles are polycrystalline. The corresponding EDS spectrum (Figure 5b) gives the signals of Cd and Te elements, confirming the existence of CdTe QDs.

**Figure 6:**
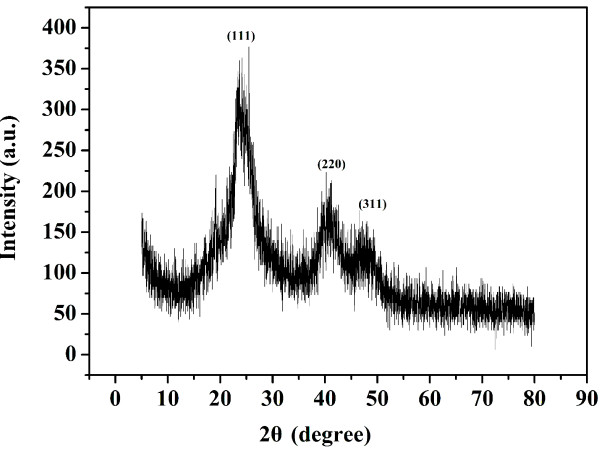
**XRD spectrum of CdTe QDs stabilized both by MPA and HPAMAM.** The excitonic absorption peak at 589 nm.

Figure 6 shows XRD pattern of the resulting CdTe QDs (the excitonic absorption peak at 589 nm). The CdTe QDs exhibit X-ray diffraction pattern consistent with cubic (zinc blende) CdTe, as represented by the broad diffraction peaks at 23.8° (111), 41.2° (220), and 48.1° (311).

**Figure 7:**
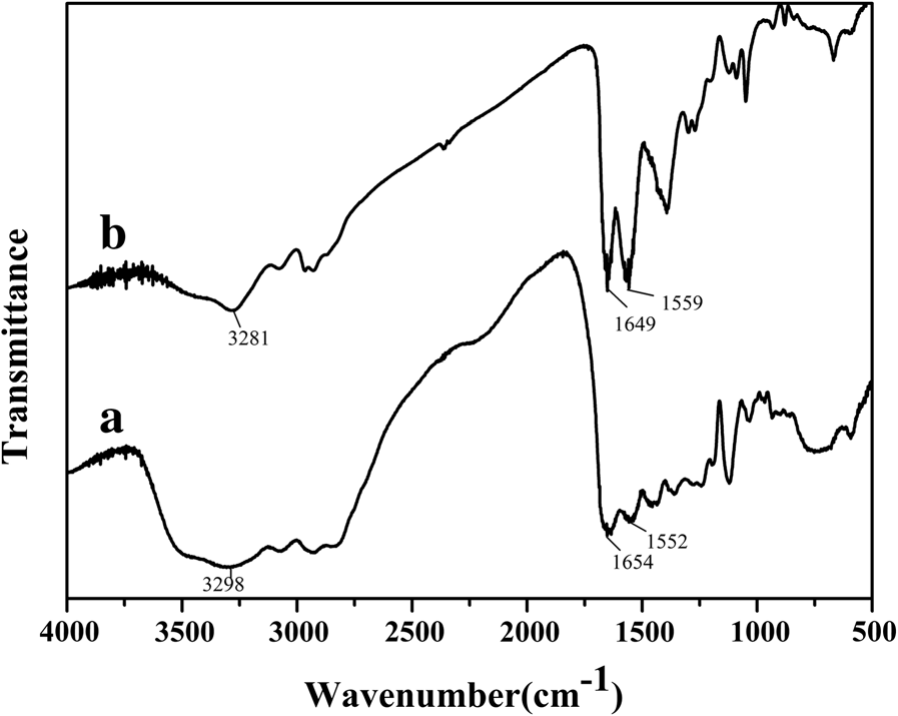
**FT**-**IR spectra of HPAMAM (a) and CdTe QDs stabilized both by MPA and HPAMAM (b).** The excitonic absorption peak at 589 nm.

Figure 7 shows a comparison of FT-IR spectra between 4,000 and 500 cm^−1^ of pure HPAMAM and CdTe QDs stabilized both by MPA and HPAMAM. The broad band at 3,298 cm^−1^ in Figure 7a is characteristic for the N-H stretching bond frequency of primary and secondary amine groups, and it has shifted to 3,281 cm^−1^ in Figure 7b. The characteristic bands assigned to amides I and II for HPAMAM are at 1,654 and 1,552 cm^−1^, while the band positions of amides I and II slightly shift to 1,649 and 1,559 cm^−1^ for the CdTe QDs stabilized both by MPA and HPAMAM. The band at 1,559 cm^−1^ in Figure 7b can also be attributed to the asymmetric carboxylate peak, which is from the MPA stabilizer.

**Figure 8:**
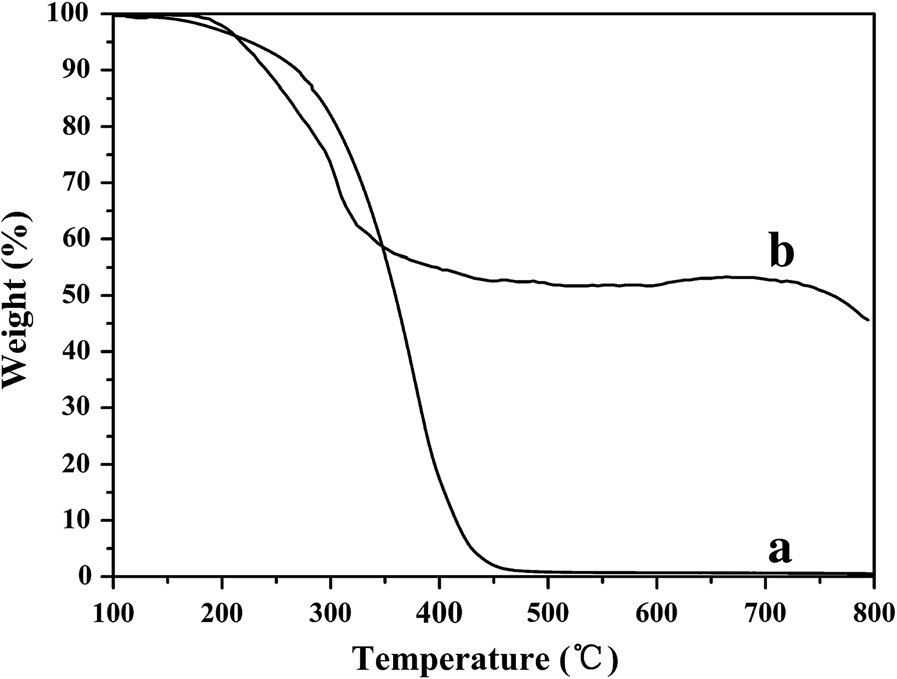
**TGA weight loss curve of (a) pure HPAMAM and (b) CdTe QDs stabilized both by MPA and HPAMAM.** The excitonic absorption peak at 589 nm.

The composition of CdTe QDs stabilized both by HPAMAM and MPA was characterized by TGA. From the TGA thermogram in Figure 8a, we can see a long temperature range from 200°C to 450°C, which is the decomposition temperature for HPAMAM. For the CdTe QDs stabilized both by HPAMAM and MPA, the weight fraction is 45.6% at 794°C, as shown in Figure 8b. This means that the content of CdTe QDs in the nanocomposites is 45.6%.

## Conclusions

In conclusion, a new strategy for *in situ* preparation of highly fluorescent CdTe QDs with MPA and HPAMAM as co-stabilizers was proposed in this paper. The resulting CdTe QDs combine the biocompatibility property of HPAMAM and the optical, electrical properties of CdTe QDs together. They also have a high QY up to 60.8%. They do not need to be post-treated and can be directly used in biomedical fields due to the existence of biocompatible HPAMAM.

## Competing interests

The authors declare that they have no competing interests.

## Authors' contributions

YS and ZM carried out all the experiments and drafted the manuscript. NC, YL, YD, XH, WD, LL, and GT participated in preparing and characterizing quantum dots. All authors read and approved the final manuscript.
